# The illegal market for sex in the United States: Insights from economic research

**DOI:** 10.1073/pnas.2512375122

**Published:** 2026-08-10

**Authors:** Manisha Shah

**Affiliations:** ^a^ Chancellor’s Professor, Department of Agricultural and Resource Economics, University of California, Berkeley, CA 94720

**Keywords:** sex market, criminalization, regulation, externalities, internet

## Abstract

The illegal market for sex in the United States generates significant economic activity yet remains understudied due to its illegality and clandestine nature. This paper synthesizes existing empirical evidence documenting aspects of market structure, impacts of regulation, and notable changes over time. Criminalization, while intended to suppress the market, often exacerbates harms by displacing activity underground, increasing violence against sex workers, and elevating disease transmission risks. Natural experiments, including decriminalization episodes in Rhode Island and policy shifts in Europe, reveal that decriminalization can reduce sexually transmitted infections and violence against women. Additionally, technological shifts-particularly the rise of online platforms due to the internet-have reshaped market organization. These findings challenge conventional legal and moral frameworks and underscore the need for evidence-based policymaking to guide effective regulation of sex markets.

Though widely practiced, sex work is primarily illegal in the United States. Globally it is estimated to bring in approximately 180 billion dollars, with the US market revenue estimated to be 14 billion dollars (1). Sex work and prostitution are terms commonly used interchangeably to describe the exchange of sexual labor for money, goods, or other resources between two mutually consenting adults. This exchange can occur in various contexts: It may be independently initiated by individuals, facilitated through a third party, or influenced by industry stakeholders. Sex work can include in-person contact with a client but can also include services that take place solely online or through the phone. The practice is as ancient as human society itself and, while frequently associated with women and girls, encompasses individuals of all genders, ages, races, abilities, and sexual orientations (2).

Although sex work is often perceived as predominantly occurring in marginalized urban areas or among underprivileged cultural groups, sex work is pervasive at all echelons of society. Media portrayals often emphasize street-based sex work, presenting it as the predominant form of prostitution; however, street-based sex work has been estimated to account for approximately 10 to 15% of total sex work participation (2). In the United States and in many other countries, the majority of the sex market has moved “indoors,” whether that be through massage parlors or brothels and/or online encounters.

The advent of the internet has profoundly transformed the commercial sex market by enabling sex workers to advertise services directly to clients without relying on intermediaries. Digital platforms now facilitate the negotiation of in-person encounters and offer tools for mutual screening, enhancing safety and autonomy for both parties. There are online encounters where an in-person service delivery is supported via an online platform for locating clients/providers and perhaps even rating them, but the sex act itself is in-person. There are also online sex services which are delivered virtually, which has preinternet antecedents in phone sex. The COVID-19 pandemic further accelerated the shift to virtual sex work, as extended lockdowns and social distancing measures prompted greater reliance on virtual forms of intimacy and income generation. The OnlyFans platform exemplifies this trend: As of 2021, more than two million content creators used the site to distribute sexually explicit material to over 130 million registered users (3). Although much of the content on platforms like OnlyFans is classified as pornography, it can also be viewed within the broader category of sex work. While both pornography and prostitution involve the exchange of sexual services for financial compensation, they differ in important respects-particularly the mediated nature of performance vs. direct physical interaction. Pornography, unlike prostitution, is afforded some level of protection under freedom of speech laws in the United States.

This article examines the legal foundations underlying the criminalization of sex work in the United States and reviews existing evidence on how different regulatory frameworks affect key outcomes. It also describes the structure of the sex market, highlights significant changes in the sex market over time, and concludes by outlining promising directions for future research.

## The Scale of the Market and Its Distribution over Space

As with other illegal markets, the covert nature of the trade makes it extremely difficult to generate reliable estimates of market size both in terms of numbers of sex workers and amount of revenue generated. Adair and Nezhyvenko (4) survey the literature on sex worker prevalence in Europe. Overall, they find there are between 500,000 and 1.1 million sex workers in the European Union (EU), depending on the methodology used, where the low-end estimate is based on extrapolating from HIV incidence, and the larger number is based on compiling reports from NGOs and other reports. This corresponds to 0.3 to 0.7% of the population of females aged 15 to 49 is a sex worker in the EU. Azam et al. (5) use data from online sex worker review websites in Belgium and the Netherlands and implement the capture-recapture model, a common method in the public health literature. They find a prevalence of 0.15 in the Netherlands and 0.18 in Northern Belgium.

While EU countries have integrated illegal drugs and illegal prostitution into their national accounts (6), similar estimates are hard to come by for the United States as the federal government does not publish official reports on prostitution (7). Potterat et al. (8) estimate the annual prevalence of full-time equivalent prostitutes in the United States to be 23 per 100,000 population based on a capture-recapture study of prostitutes in Colorado Springs, though it has been argued this study likely underestimates the number of sex workers (9). Fondation Scelles (10) estimates there are 1 to 2 million sex workers in the United States, which represents about 0.8 to 1.6% of the female population in the United States. While reliable estimates of the prevalence of sex workers in the United States are not currently available, the 1 million number is likely the closest estimate.

In accordance with the high prevalence of female sex workers, the dollar value of the global sex market is believed to be substantial. In the European Union, Adair and Nezhyvenko (4) sum up national accounts and conclude that the market was worth 23 billion euros in 2010, or $35 billion in 2024 dollars, adjusting for inflation and currency exchange.^*^ In 2017 in the United States, nominal GDP rose by more than 1% when illegal activity was tracked. Illegal prostitution adds $10 billion to measured GDP which is about 0.05% of GDP in 2017 (11). However this is likely an underestimate as the study proxies for price and quantity with legal care worker wages and sex worker arrests, both of which are likely to produce underestimates (12, 13). While national level data in the United States is sparse, Dank et al. (14) provide estimates of the US market size at a subnational level in seven US cities. In 2007, in Atlanta, Dallas, Denver, Miami, San Diego, Seattle, and Washington DC metro areas, the authors estimate that the total revenue of the sex market is $975 million, or about 1% of all expenditures.

Sex worker prevalence in lower-and-middle income countries (LMICs) tends to be higher on average (15). Some of this is likely due to fewer employment opportunities for women and overall worse outside options. In Latin America, 0.81% of all adults, male and female, ages 15 to 49, are sex workers (16). Laga et al. (17) study female sex worker prevalence in Sub-Saharan Africa and estimate an overall prevalence of 1.1% for females ages 15 to 49, which ranges from 0.32% in Malawi to 2.8% in Burundi. In addition, some low-income countries are destinations for international sex tourism which increases the size of the domestic sex market. For example, in Thailand, one of the most common destinations for sex, sex work accounts for 2 to 4% of GDP (18), which seems quite high.

While the majority of this literature focuses specifically on female sex workers and male clients, there is also a need to estimate the share of the supply of sex workers that is male and/or transgender. Estimates of the share of sex workers that are male tend to fall between 5 and 15% (4, 19, 20), though many of these studies are highly local and based on convenience samples. As a percentage of the total transgender population in the United States, the 2008–2009 National Transgender Discrimination Survey found that 10% of respondents had ever traded sex for money (21). The gap in our understanding of male and transgender sex work is an additional challenge for estimates of overall market size (e.g. refs. 11 and 22).

## Primary Basis for Illegality

Sex work is illegal in the United States, except for a few rural counties within the state of Nevada and a recent change in Maine.^†^ Sex work is illegal because of state laws, not federal laws. It is therefore exclusively the domain of the states to permit, prohibit, or otherwise regulate commercial sex, except insofar as Congress may regulate it as part of interstate commerce with laws such as the Mann Act. The Mann Act of 1910 criminalizes the transportation of women or girls for the purpose of prostitution or other immoral purposes (25). In 1913, in Hoke vs. United States, the Supreme Court ruled that prostitution should be regulated at the state level, though Congress still had jurisdiction to regulate interstate travel for the purpose of prostitution. Nevada initially passed laws to rein in the trade but changed course in 1971 and began to permit it as a regulated activity in certain parts of the state (26).

The rapid decline in “red light districts” and legal brothels in America starting from the beginning of the 20th century was in large part due to the growing advocacy of “morality” groups. The basic argument against prostitution was personal and moral—that irregular sex relations were a breaking of God’s Law. Increasingly, advocates came to view prostitution as a big business that preyed on vulnerable women, often very young or recent immigrants (25). There was also fear they would spread immorality and corrupt others with their trade. Various social and criminal problems were also associated with prostitution, including drug use, theft, and disease transmission.

The criminalization of sex markets in the United States was largely due to moral repugnance, the idea that even if the parties directly involved benefit from the trade, because of moral concerns, individuals and societies oppose that such a transaction occur (27). In a 2012 poll where Americans who opposed legalizing prostitution were asked for their primary reason, 44% opposed it simply because they view it as morally wrong, 25% feared it will contribute to the spread of STIs, 15% said it violated their religious beliefs, and 9% felt it undermined marriage (28). Interestingly, support for legalizing prostitution in the United States has grown recently, from 38% in 2012 to 48% in 2015 (28, 29), and has remained steady into the 2020s (30). A survey of young Americans in late 2019 finds that 64% of respondents believe that prostitution is not innately a social evil (31).

There is also a primarily Western feminist case for criminalizing the buyers of sex. Freeman (32) takes issue with the notion that prostitution is a victimless crime, and argues that regulations should address “the role prostitution plays in the subordination of women.” This view of sex workers as victims motivated the 1999 enactment of a new regulatory approach in Sweden known as the Nordic model, where male buyers are criminalized and female sellers are not (33).

The other major push for illegality in the United States is related to the negative externalities associated with sex markets, whether that be disease transmission, drug use, violence, declining real estate prices, etc. For example, concerns may arise about the negative effects of commercial sex on the quality of life in neighborhoods where it is prevalent (27). Economic theory suggests that criminalizing the market should keep the market size relatively small as criminalization increases costs to entry and imposes costs on both sex workers and clients already participating in the market. If negative externalities like disease transmission and violence are an increasing function of the size of the market (or total sex occurring), then criminalization should decrease the negative externalities. However, if criminalization encourages riskier behaviors among continuing participants and/or entry of new riskier participants, this could exacerbate the negative externalities.

## Primary Harms Created by Illegality and Illegal Markets

Causally measuring the primary harms created by illegal sex markets is often difficult due to lack of variation in policy. When jurisdictions do change policy it usually happens at the national level, so the empirical question becomes what is the right counterfactual for an entire country? Much of the causal literature in this space has exploited natural experiments to estimate the causal impacts of criminalization (or decriminalization). The decriminalization of sex work “refers to the removal of criminal penalties for the buying and selling of sexual acts, specifically those categorized as prostitution” (34). A decriminalized regime is one where sex work is allowed because there is no law preventing it. This is distinct from legalization, where a specific law states that prostitution is allowed, provided participants comply with certain regulations.

A review of some of the empirical evidence on illegal sex markets, focusing on causal literature when available, is presented next.

### Pushing the Market Underground and into The Street?

A common theme in the literature on sex work is the two-tiered nature of the profession, the outdoor street market and the indoor market. The street market is riskier and pays less across the globe. In the United States, while indoor workers earn around $4,000 per week seeing about four clients (12, 35, 36), street workers earn closer to $500 per week seeing around seven clients (37). In a survey of sex workers in England, 44% of outdoor workers report experiencing violence from a client, compared to 18% of indoor workers (38). The mortality rate from homicide for street prostitutes is 204 per 100,000 making it the most dangerous profession for women (39).

This presents a challenge for regulating sex work. While there are real harms associated with the indoor sex sector, attempts to regulate/criminalize these activities risk pushing the market underground and/or onto the street which in turn may result in more negative externalities. Cameron et al. (40), who study the criminalization of brothel-based sex work in Indonesia show that immediately after criminalization, the size of the market shrinks. However, five years after criminalization, the market has rebounded to its original size, though is less organized and more underground.

Gertler and Shah (41) show that this displacement effect can work both ways. Studying the enforcement of the carnet system, an occupational license required of sex workers in Ecuador (and in much of Latin America), they find that high levels of enforcement, as measured by police raids in bars and brothels, result in more (unlicensed) sex workers shifting to the riskier street sector. In cities with more street-level police visits, entry into the indoor sector becomes more common. The authors conclude that efforts to regulate the indoor market can backfire if they are not paired with measures to keep workers off the street.

### Disease Transmission and Criminalization.

Disease transmission has been associated with sex markets since syphilis outbreaks in the late 15th century. By the 16th century, the disease was seen as “evidence of moral failure” within European society (42). Centuries later the AIDS epidemic of the late 20th century reignited a fear that sex work inevitably leads to an increase in sexually transmitted infections (STI) and HIV, especially in low-income countries. The link between sex work and disease transmission continues to be a source of concern, though more so in low-income settings which still have high rates of new HIV/AIDS cases. UNAIDS (43) classifies sex workers as a “key population” in the effort to fight HIV/AIDS, since their rate of infection is 2.7%, compared to a general population prevalence of 0.7%.

Cameron et al. (40) provides causal evidence on the impact of criminalizing sex work on the spread of STIs in East Java, Indonesia, using a natural experiment where sex work was criminalized in one district (basically overnight) while remaining legal in neighboring areas. Employing a difference-in-differences approach, the study analyzed panel data collected from sex workers and clients before and after the criminalization. The findings reveal that criminalizing sex work leads to a 27.3 percentage point (or 58%) increase in STI rates among sex workers, measured by biological tests. The primary reasons for this increase include reduced access to condoms, increased condom prices, and a rise in noncondom sex under criminalization. The sex workers also report lost income and lower happiness. In addition, children of women from criminalized worksites are adversely affected—they have less money for school and are more likely to start working to supplement household income.

One might argue that these impacts observed in a middle-income country like Indonesia might not carry over to high income regions like the United States. While there is no natural experiment on criminalization in the United States, there is some interesting evidence on decriminalization. Cunningham and Shah (44) examine the causal impact of decriminalizing indoor prostitution in Rhode Island in July 2003, which occurred due to an unexpected judicial decision that remained in place until November 2009, when indoor prostitution was recriminalized. During this six-year period, Rhode Island became the only US state with fully decriminalized indoor prostitution, while street prostitution remained illegal.

The research shows that the decriminalization led to a notable decrease in total arrests of sex workers, an expansion of the indoor prostitution market, increased advertising for indoor services, and lower transaction prices in Rhode Island. Importantly, the study finds strong evidence that decriminalization resulted in a 40% reduction in the incidence of gonorrhea among women in the state of Rhode Island using CDC data. The authors suggest that the reduction in STIs is due to decriminalization diluting the“core group” of high-risk sex workers by attracting lower-risk individuals into the market, thereby reducing the overall risk of STIs (45, 46). Additionally, sex acts become less risky after decriminalization, with decreases in anal and vaginal sex without condoms and increases in oral sex with condoms (44).

These results are consistent with a meta-analysis of quantitative and qualitative studies examining the associations between sex work laws and sex workers’ health. Platt et al. (47) find that across 12,506 participants from 11 studies, sex workers exposed to criminalization and repressive policing practices are on average at increased risk of infection for HIV and STIs. The WHO also finds that decriminalizing sex work worldwide could lead to a 46% reduction in new HIV cases among sex workers over the next 10 y (48). While studies of criminalization in modern contexts tend to find an increase in the spread of disease, there is evidence that government regulation can help reduce the spread. A policy of registration, testing, and forced isolation of sex workers reduced the spread of syphilis in Victorian England (49). Likewise, mandated regular testing in the legal Nevada brothel sector contributes to a low spread of disease (24).

### Violence Against Women and Criminalization.

Sex workers face some of the highest rates of violence against women across the globe. The prevalence of physical and/or sexual violence female sex workers experience is between 40 to 70% in a one year period (50). When sex work is criminalized, police might not be as motivated to protect sex workers from harm and sex workers are less likely to request help. Rape is also associated with an increased likelihood of acquiring sexually transmitted infections and/or HIV, so the prevention of sexual violence is also related to disease transmission (50). Advocates for criminalization argue that countries which decriminalize or fully legalize sex work will see an increase in violence against sex workers (51). However, these claims lack empirical support.

Cunningham and Shah (44) show that in Rhode Island, when indoor sex work is decriminalized, reported rape offenses decrease 31 to 34% from 2004 to 2009. Bisschop et al. (52) evaluate the opening of legal street prostitution zones in twenty-five cities in the Netherlands and find that legal prostitution zones are associated with a 30 to 40% decrease in sexual abuse and rape. Nguyen (53) finds reducing costs to opening (illegal) massage parlors leads to as much as a 28% decrease in all rape offenses in California. Moreover, there is strong evidence suggesting that criminalizing prostitution may increase the risk of violence and victimization for sex workers (54). Many sex workers report being reluctant to report crimes against them due to fears of being blamed for their victimization or arrested for engaging in illegal activity (55). In South Africa, for instance, the criminalization of sex work was found to exacerbate rates of sexual violence against workers (56). Findings from various studies suggest that the criminalization of prostitution likely raises the vulnerability to violence by clients and makes it less viable for victims to report crimes to police if they occur, thereby increasing the risk of victimization (57).

## Extent of Enforcement and Regulation of Sex Work

Each state in the United States has established its own laws regarding the criminalization of prostitution, penalties for customers, penalties for pimps, and penalties for brothel owners. In most states, prostitution is considered a misdemeanor in the category of public order crime, a crime that disrupts the order of a community. Penalties for a first prostitution charge can involve misdemeanor jail sentences or fines up to $10,000. For repeat offenders, penalties can include felony prison sentences and fines up to $25,000 depending on the state. Penalties for pimps and brothel owners are primarily classified as felonies with heavy fines and lengthy prison sentences, but not all states are as punitive (58). The variation in state penalties for prostitution and the associated behaviors reflect the differing local customs and traditions. Additionally, these variations reveal there is disagreement on whether and how to criminalize prostitution and the related offenses across the state jurisdictions in the United States.

**Fig. 1.**
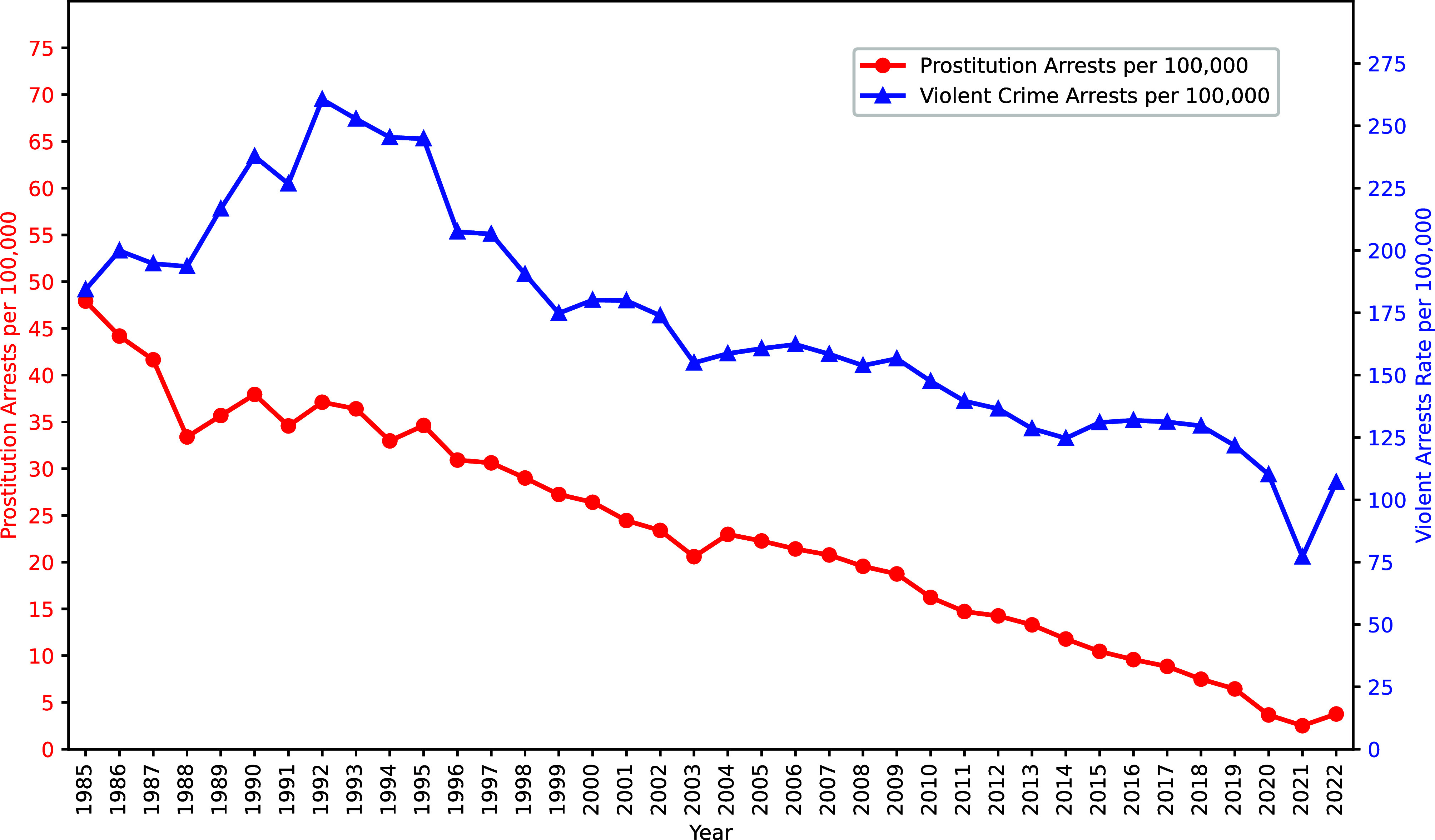
US arrest rates for prostitution and violent crime (per 100,000), 1985–2022. *Notes.* This figure shows the trend in the arrest rate for prostitution from 1985 to 2022, compared to the arrest rate for violent crimes. Violent crimes as defined in the Uniform Crime Reports are murder and nonnegligent manslaughter, rape, robbery, and aggravated assault. The arrest rate is the total number of arrests divided by the US population in that year. *Source.* Arrest counts from the FBI Uniform Crime Reporting System (UCR) (59). Population data from FRED (60).

Prostitution arrests have been decreasing over time in the United States. Fig. 1 plots the arrests for prostitution in the United States from 1985 to 2022 showing that the arrest rate has fallen dramatically from 50 per 100,000 in 1985 to less than 5 per 100,000 in 2022. Though the rate has been falling for all sex workers, the prostitution arrest rate for Black sex workers remained about four times higher during the period 2000–2021. Fig. 1 also shows that violent crime arrests have been falling over time at a similar rate since the 1990s to prostitution arrests. Even as arrests have fallen by 88% from 1988 to 2022, the percentage of Americans who report participating in paid sex in the past year increased from 0.3% in 1988 to 0.9% in 2022 (61). Studies also show suggestive evidence that the indoor sex sector has grown over the past 25 y (e.g. refs. 62 and 63). Therefore, the decline in arrests is not due to shrinking supply or demand in the sex market. Fig. 1 is likely explained both by a large-scale displacement of street prostitution by indoor prostitution (12), since street prostitutes are far more likely to be arrested (64, 65), as well as shifting priorities by law enforcement.

## Structure and Nature of Enterprises

Sex work is an industry generally characterized by small supplier organizations, even to the extent that most suppliers are independent and self-employed (12, 66). In its simplest form, sex work requires no capital, has no monetary cost to entry, and does not require a highly specialized skill set. As such, competition is strong between workers and supplier organizations, and sellers typically have little market power (37, 67). The ultimate organizational structure of enterprises depends on the modality of sex work in question. The main locations that sex workers offer services are the street, brick and mortar locations like brothels or massage parlors, and online as freelance or agency workers. Prostitutes who find clients on the street or online are often able to work alone (12, 68). Sex workers based in brick and mortar locations are much less likely to be self-employed.

### Street Sex Work.

While street prostitution looms large in the public imagination, it is actually a small portion of the total market in the United States. Estimates find that between 10 and 30% of sex workers work on the street (65, 69, 70), though the challenge of observing this market makes estimation difficult.

Compared to the indoor sex sector, prices tend to be lower on the street (68, also see Table 1). While emotional connection is an important component of some sex work, transactions on the street tend to be more transient, focused only on physical sex and typically lasting 10 to 15 min (64). In areas known for street sex work, potential clients can signal interest to a seller by driving by slowly and making eye contact. Workers must quickly ascertain whether the client is a threat for violence or an undercover police officer. Many street workers use nothing but their intuition to determine whether a client is risky (85). In contrast, indoor workers will spend considerable time vetting clients and often get references from other sex workers (65, 86). This rushed vetting process is often cited as a reason why transactions on the street have such a high rate of violence (62).

**Table 1.** Sex worker and non–sex worker wages across the globe

Source (1)	Group (2)	Wage (2024 dollars) (3)	Comparison wage (4)
Gertler et al. (71)	Sex workers in Morelos and Michoacan, Mexico	$14/h	34% more than same-age women
Levitt and Venkatesh (37)	Street sex workers, Chicago, US	$39 to $47/h	Four times wages in other activities
Edlund et al. (72)	Escorts, US	$410/h	$27/h for US women (73)
Cunningham and Kendall (74)	Freelance sex workers, US	$544/h	$27/h for US women (73)
Robinson and Yeh (75)	Sex workers, Kenya	$14/d	Four times other women in the area
Cunningham and Kendall (12)	Online sellers, US	$3,850/wk	$1,060/wk median for all women over 16 (76)
Gertler and Shah (41)	Brothel sex workers, Ecuador	$10/h	$2 to $4/h for women, assuming 160 h per month (77)
Gertler and Shah (41)	Street sex workers, Ecuador	$7/h	$2 to $4/h for women, assuming 160 h per month (77)
Morris (36)	Legal brothel sex workers, Nevada	$4,047/wk	$962/wk for Women in Nevada (78)
Brooks-Gordon et al. (22)	Street sex workers, London	$33,000/y	$54,460/y for women in London (79)
Brooks-Gordon et al. (22)	Off-street sex workers, London	$107,714 to 335,373 after expenses	$54,460/y for women in London (79)
Egger and Lindenblatt (80)	Online sex workers, Germany	$262/h	$24 for women in Germany (81)
Drucker and Nieri (70)	Online sex workers, US	$236 to 524/h	$27/h for US women (73)
Islam and Smyth (82)	Sex workers, Bangladesh	$187 to 222/mo	Top 1.5% of female incomes
Cunningham and Kendall (35)	Indoor sex workers, US	$2,936/wk	$1,060/wk median for all women over 16 (76)
Weitzer (64)	Escorts, US	$224 to 6,733/h, plus gifts in some cases	$27/h for US women (73)
Manian (83)	Sex workers, Dakar, Senegal	$153/mo	$57 to $149 for domestic servants (84)

This table presents collected estimates of wages of sex workers. Column 1 gives the source, column 2 describes the group of sex workers for which the source estimates wages, and column 3 gives the wage in 2024 dollars. Inflation adjusted prices are calculated using the CPI inflation calculator from the BLS. Dollars are converted to November 2024 from the date of data collection or if that is unavailable, the date of publication. Column 4 gives a reference point for what a sex worker in a given setting might expect to make in a different profession. Where possible, this reference point is taken from the same source as the earnings estimate. Otherwise, an independently sourced comparison is given.

While many street prostitutes work alone, it is also common to work for a pimp. Pimps can add value by sourcing clients, providing protection, and providing other business services like a hotel room. In Chicago, prostitutes who work with a pimp see fewer clients, charge higher prices, and are less likely to be arrested (37). In a survey of 73 pimps from eight US cities, two thirds reported providing transportation and housing for their employees, and many also paid for clothes, advertisements, and legal fees, among other expenses (14). However, many street workers do not report significant benefits from working with a pimp, and there are alarmingly high rates of coercion and abuse (14, 68). Estimates of what proportion of street workers are employed by a pimp can range widely across locations, from 10 to 80% (85, 87–89). Pimp-run operations also tend to be small and independent of other pimps or organized crime, with most pimps having 2 to 8 employees (14).

### Brick and Mortar Businesses.

Brick and mortar locations like brothels and massage parlors tend to be larger operations where sex workers are often employees. Legal brothels operate across the state of Nevada, employing about 500 workers across roughly 25 to 30 establishments (24). There is also an unlicensed brothel sector across the United States that is not well understood due to its secretive nature. Much of the brick and mortar sector in the United States is massage parlors. Illicit massage parlors coexist in the world of legal massage parlors, making it hard to differentiate the two. Illicit massage parlors in particular comprise a large portion of the overall prostitution sector, with estimates of total revenue ranging from 2.5 to 4 billion dollars (90–92). Clients typically pay a fee for the base massage, and then an additional fee or tip for sex. Massage parlors differ in how they split fees and tips between house and worker. In cases where workers receive very little of the base fee, there is more pressure to provide sexual services for tips (93). Some parlors are part of larger networks while others operate as stand-alone operations.

Illicit massage parlors are usually small businesses. Only seven percent of businesses have more than 20 employees (53). The average massage parlor connects to at least one other massage parlor, as well as other businesses like nail salons that can be used to launder money (90). Massage parlors can share information about troublesome clients and rotate employees among each other, which allows massage parlors to continually present new faces to customers while preventing exploited workers from making strong personal connections or establishing their footing in one location (14, 93).

There is a concern that illicit massage parlor workers are being trafficked (e.g. refs. 92 and 94). Illicit massage workers can overstay tourist visas, which are often acquired with the help of a broker or smuggler for a fee (90). These migrants, frequently facing financial pressure and lacking fluent English or a work permit, often view illicit massage parlors as the best of very limited employment options (93). While most workers in these massage parlors are voluntary, there are cases of outright coercion, and it is still an open question as to how many are being trafficked and how connected the traffickers and massage parlors are.

### Online Independent and Agency Sex Work.

The most prevalent form of sex work in the United States today is an indoor, internet-mediated market where clients can find a sex worker through an agency or online listing, and transactions take place at a location provided by the client (outcalls) or worker (incalls). Escort agencies originated as brothels that sent workers out to clients and gradually evolved to their current online form. For a cut of a worker’s revenue (typically 40 to 50%), an agency will source and screen clients, and offer other forms of support like education for novice workers (95). However, the introduction of online platforms to match buyers and sellers has atomized the market, allowing many sellers to go independent.

Where outdoor sex work is almost entirely physical, indoor workers often describe emotional connection as a core part of the service they provide. This emotional component also features prominently in advertising for sex work, where it is referred to as a girlfriend or honeymoon experience (95). Workers with a college degree tend to have longer sessions with clients where nonsexual services such as companionship play a larger role (35). While outdoor transactions tend to last 10 to 15 min (64), indoor transactions last longer, at two hours on average, and are even longer for college-educated workers (35).

Historically, it was difficult for sex workers to source clients, especially without a physical presence in a bar or outdoor stroll, but the advent of online platforms has dramatically reduced these search costs, resulting in a thickening of the market for escort services. The Craigslist Erotic Services board is associated with an increase in total market size and a shift away from agency work and toward independence (62). In a 2008–2009 survey, 93% of workers listed in the popular review website The Erotic Review are independent, though this could be an overestimate if independent workers are more likely to respond to the survey (12).

Today, internet-mediated sex work is ubiquitous, and while determining the size of the market is difficult, it has been estimated to comprise 80% of the total sex market in the United States (96). While prostitution is illegal in the United States, common carrier laws created a loophole whereby platforms were not liable for hosting advertisements for sex. Today, sex workers advertise their services widely on escort sites, personal websites, and covertly on social media. Some of these platforms like OnlyFans are officially based outside the United States (97).

### Earnings Profile and Wage Premium for Sex Work.

In a typical labor market with low barriers to entry, low skill requirements, and strong competition, wages would be low. Edlund and Korn (13) consider the puzzle of why prostitutes earn so much and argue the phenomenon is driven by reduced marriage prospects for sex workers. Arunachalam and Shah (98) argue that the premium to sex work is likely due to a compensating wage differential which accounts for all the risks sex workers face on the job, including disease, violence, and arrests.

Table 1 demonstrates that in the United States and around the world, sex workers can earn a significant amount of money, especially when compared to non–sex workers with similar demographic characteristics. Column 1 lists the study, column 2 defines the type of sex work, column 3 summarizes the wages (in November 2024 dollars), and column 4 offers a reference point for what similar workers in other fields can expect to earn. Where a reference point is provided in the paper, it is listed in column 4. Otherwise, a separately sourced reference is listed. The table generally shows a large premium to sex work. Street workers in Chicago surveyed by Levitt and Venkatesh (37) can earn close to $50 dollars per hour, four times the wages they would earn otherwise. Indoor sex workers can earn considerably more, about $400 to $550 per hour on average, or $3,850 per week (12, 72, 74). This compares to a median of $27 per hour or $1,060 per week for all women in the United States. Robinson and Yeh (75) report sex workers in Kenya can earn $14.13 per day, about four times what other female daily income earners make in the area. In Ecuador and Mexico, Arunachalam and Shah (98) estimate a wage premium of about one-third for sex workers by regressing the earnings of survey respondents on whether they are a sex worker. Regardless of the country, it appears sex workers are earning a premium.

Sex work is characterized by a large premium for youth and physical attractiveness (99). In this respect, it parallels professions such as professional sports, where earnings peak early in life and decline with age. As in the case of athletes, many sex workers may face a significant drop in income as they age, potentially leading to reduced consumption in later years (100). This trajectory contrasts sharply with most conventional career paths, where earnings typically rise over time through accumulated experience, promotions, and skill acquisition. Sex work career longevity varies significantly, with many individuals having short-term engagements while others maintain longer careers. A longitudinal study of sex workers followed for fifteen years found that the majority were still in the sex industry and had sold sex for a mean of 13.6 y (101).

## Notable Changes and Innovation over Time

### Impact of Internet Regulation on Sex Work.

One of the most notable changes in the sex market, especially in high income countries is the growth of the online sex market. The widespread adoption of the internet has upended how sex work is advertised (44, 102, 103) and delivered (104). As such many sexual services provided today in the United States require some level of online involvement (102). The addition of the internet to sex work has provided sex workers with more autonomy when it comes to protecting their safety. Sex workers can advertise on classified websites, sex worker registries, personal websites, dating apps, and social media platforms. Where information on prostitutes was once difficult to come by, rating websites like The Erotic Review allowed customers to exchange information on sex workers and provided another avenue for sex workers to advertise their services. These review forums also serve as an enforcement mechanism to ensure advertisements are truthful (103). Sex workers also use websites to share information about clients, vet clients, receive peer support, and network (64, 67).

Regulators and law enforcement were caught off guard by the changes in use of technology. Many of the legal developments in recent years have been driven by the government coming to terms with the new face of online prostitution (102, 105). The 1996 passage of the Communications Decency Act attempted to regulate the spread of indecent content on the internet. However, lawmakers were concerned that the bill would be onerous for platforms that hosted and moderated user content, so they created a carveout in Section 230, shielding platforms from liability for user-created content. Section 230 has since become a bedrock piece of internet regulation (105, 106), and an unintended consequence is that platforms were not liable for prostitution advertisements on their websites(86, 102).

Regulators passed FOSTA-SESTA in 2018, an antitrafficking law which weakened Section 230 by expanding federal criminal liability for sex trafficking and made internet platforms liable for sex trafficking offenses (105). Websites like Backpage immediately shut down due to FOSTA-SESTA (102). When online platform Backpage shut down in 2018, a survey of 98 sex workers recruited online through social media and activist networks showed that 73% of respondents felt more economically unstable, and 33.8% reported higher incidents of violence from clients. In the aftermath of these closures, sex workers reported reduced income and a reduced ability to screen clients (107).

While the intent of FOSTA-SESTA was to fight human trafficking, little is known about the incidence of trafficked sex workers in online spaces, so it is difficult to estimate the effectiveness of the law. The reduced presence of prostitution websites, as well as the COVID-19 pandemic, coincided with the rapid rise in popularity of OnlyFans, an online platform for digital delivery of pornography and sex work. An advantage of OnlyFans compared to traditional pornography is the opportunity for communication and emotional connection between creator and user (104). OnlyFans may represent a significant new competitor to the indoor prostitution industry offering new employment opportunities to sex workers. While most creators earn less than $2,000 per year, top accounts can take home millions (104, 108). The extent to which websites like OnlyFans is crowding out demand for traditional sex work, and the effects this might have on earnings, violence, and STIs is unclear at this time.

### Changing Global Regulatory Environment.

Another big change recently is related to regulatory frameworks across the globe. The Nordic Model (also known as the End Demand Model) has quickly spread to multiple countries in the Global North. The Nordic model was first implemented in Sweden in 1999, where prostitution had previously not been regulated. Sweden banned the purchase of sex, aiming to “address the root cause of prostitution and trafficking in beings: the demand, the men who assume the right to purchase persons for prostitution purposes” (33). Since then, the End Demand model has been adopted by Norway, Iceland, Canada, Northern Ireland, France, Israel, and Ireland (109). In 2023, Maine joined this growing list of jurisdictions in the global North employing the Nordic model of prostitution regulation.

Backus and Nguyen (110) study the effect of banning the purchase of sex in Northern Ireland and find that the law reduced transaction quantity and price at first, consistent with a decrease in demand. However, the market begins to rebound, such that three years after the passage of the law, there is little evidence of reduced demand. This is consistent with findings from other studies that show the Nordic Model has not changed the overall size of the sex market as much as it has displaced workers (109). Giusta et al. (111) study how demand changed in the United Kingdom before and after the passage of a series of laws which criminalized the buyer soliciting sex in public places. Their findings suggest that the profile of clients changed to riskier ones which is likely nonoptimal from a public policy/safety perspective. This finding aligns with the theoretical model of criminalization put forward by Lee and Persson (112), who predict that the Nordic model will induce “negative selection” effects where violent clients will be more likely to stay in the market.

Banning the purchase of sex also displaces demand to international sex tourism destinations. Exploiting the staggered adoption of the Nordic model in four countries, (113) show banning the purchase of sex causes an increase in registered tourist visits to Thailand and the Philippines (consistent with findings in ref. 114).

While the legal status of prostitution has stayed mostly static in the United States, there is some movement for change. Legislators in New York have introduced competing bills to decriminalize prostitution and to implement the End Demand model. It remains to be seen whether change will spread beyond Maine, and what the effects of the changes might be.

### Sex Work and Sex Trafficking.

Conflation of sex work and sex trafficking is rife in the policy and academic discourse as well as in the media. However, a critical distinction must be made between sex work and sex trafficking. The defining difference lies in agency: Sex trafficking involves coercion, fraud, or force, often with a third party profiting from exploitation, whereas sex work involves some element of choice. In the United States, all commercial sex involving minors is legally classified as trafficking, irrespective of coercion, which can be problematic. Reliable data on the prevalence, revenues, and dynamics of sex trafficking are also lacking. The overlap between consensual sex work and trafficking remains poorly understood, yet this distinction is crucial for effective policy design. Policies aimed at reducing trafficking, such as FOSTA-SESTA, may have unintended negative consequences for consensual sex workers. Conversely, policies targeting sex work may affect trafficking in complex ways. While some studies suggest that legalization of sex work correlates with higher rates of trafficking (115, 116), others report a positive relationship between criminalization and trafficking (117). Recent theoretical work also highlights that the effects of policy interventions depend heavily on implementation and local context (118). However, much of this empirical literature has been criticized for using unreliable data, lacking a causal identification strategy, and possible violations of standard identification assumptions for cross-sectional analysis (118, 119). Given the importance of the issue, there is a continuing need for careful empirical research in this area.

## Research Prospects and Limitations

Despite the considerable size and economic relevance of commercial sex markets, economic research in this area remains limited. This gap is partly attributable to the scarcity of reliable data and the clandestine nature of the market. Substantially more empirical and theoretical work is needed to understand the size, structure, and dynamics of these markets—both within the United States and globally—and dedicated funding should be allocated to support such research. The lack of reliable estimates on the size of the US sex market is problematic for policy and research. Also, much of the existing literature focuses on cisgender women as providers of sex and cisgender men as buyers, reflecting the dominant market structure. Yet, emerging markets involving men who have sex with men and transgender individuals are growing and also remain significantly understudied. Second, a critical distinction must be made between sex work and sex trafficking, and a better understanding of the intersection of the two both in terms of magnitude and impacts of policies is needed from the research community.

Last, as sex markets increasingly move indoors and online, further investigation is warranted into the implications of this shift. If online platforms reduce risks and negative externalities, this raises important questions about the efficacy and desirability of continued criminalization. Nevertheless, the potential expansion of the market under decriminalization and the general equilibrium effects of such policy changes remain uncertain. Shifting from moralistic to evidence-based approaches holds the potential to reduce harm to sex workers and enhance overall societal welfare.

## Data Availability

All study data are included in the main text.

## Footnotes

^*^Inflation adjustment in Euros via the Harmonised Index of Consumer Prices (HICP). Currency exchange is based on January 2024 rates from the European Central Bank.

^†^In Maine since 2023, working as a sex worker is legal but the purchase of sex remains prohibited (23). In Nevada, brothels have existed since the old mining days of the 1800s and were first licensed in 1971. Prostitution is legal in 10 of Nevada’s 17 counties in smaller, more remote places (24).
